# SwMrNet: A Multi-Target Tissue Segmentation Method for Robust and Accurate Clinical Knee Diagnosis Assistance

**DOI:** 10.3390/bioengineering13070784

**Published:** 2026-07-08

**Authors:** Li Li, Yuwen Xing, Wenyi Xiong, Shenghui Liao, Beiji Zou, Xiangxiang Sun, Liqiang Zhi

**Affiliations:** 1 School of Automation, Central South University, Changsha 410083, China; lilicsu@csu.edu.cn; 2 Department of Knee Joint Surgery, Honghui Hospital, Xi’an Jiaotong University, Xi’an 710061, China; yuwenxing110@163.com (Y.X.); zeltasun@163.com (X.S.); 3 School of Computer Science and Engineering, Central South University, Changsha 410083, China; xwycsu@csu.edu.cn (W.X.); lsh@csu.edu.cn (S.L.); bjzou@csu.edu.cn (B.Z.)

**Keywords:** knee osteoarthritis, multi-target segmentation, knee joint, feature fusion, OAI-ZIB, explainability

## Abstract

With the acceleration of global population aging, the incidence of knee osteoarthritis (KOA) has risen significantly, placing unprecedented pressure on healthcare resources and creating an urgent need for automated segmentation technologies to enhance clinical diagnostic efficiency. Therefore, this paper proposes a novel multi-target tissue segmentation network for knee joints, SwMrNet, which integrates improved Swin Transformer units and a proposed multi-scale residual module within the decoder to enhance both segmentation accuracy and robustness. Firstly, a sliding-window mechanism is used to iteratively exchange feature information, allowing for the extraction of global tissue features. Then, features are extracted at multiple scales, with residual connections preserving the fine details of each tissue type. Through the repeated fusion of global and local features, the SwMrNet segmentation performance and robustness are significantly enhanced. Finally, the proposed model was evaluated on a public knee MRI dataset and a local clinical knee MRI dataset. On the public dataset, the model achieved a Dice score of 98.2%, with Dice scores for all segmented tissues exceeding 94%. On the local clinical dataset, the model showed visually consistent segmentation results, suggesting its potential as an efficient multi-tissue segmentation tool for automated knee joint analysis and auxiliary clinical assessment.

## 1. Introduction

Knee osteoarthritis (KOA) is a globally prevalent degenerative joint disorder characterized primarily by pain and restricted mobility, which severely impairs patients’ independence and quality of life [1,2,3]. As of 2024, the number of KOA patients in China has surpassed 120 million, with a prevalence rate of 8.1%. With an aging population, the demand for total knee replacement surgeries is projected to increase by 673% by 2030 [4,5], imposing substantial burdens on healthcare resources and financial systems. Currently, there is no definitive cure for KOA, largely due to the lack of a comprehensive understanding of its underlying pathological mechanisms. Recent studies have also investigated knee joint acoustic signals as a low-cost and noninvasive approach for assessing cartilage degeneration, demonstrating the broader development of artificial intelligence-assisted knee assessment beyond conventional imaging-based methods [6,7]. However, the present study focuses on multi-target tissue segmentation in knee magnetic resonance imaging.

In clinical practice, magnetic resonance imaging (MRI) plays an important role in KOA assessment owing to its superior soft-tissue contrast compared with computed tomography (CT) [8,9,10,11]. The high spatial resolution of MRI enables precise differentiation of intra-articular soft-tissue structures such as cartilage, ligaments, and menisci, making it an essential modality for detecting localized structural changes within the knee joint [12]. Accurate segmentation of anatomical components such as the femur, tibia, patella, and ligaments from MRI scans is critical for the early identification of KOA. This facilitates not only the prediction of disease progression but also timely clinical intervention before irreversible pathological damage occurs [13,14]. Despite its importance, tissue segmentation remains a significant challenge [15,16]. Traditional manual segmentation methods depend heavily on clinician expertise, rendering the process labor-intensive, time-consuming, and prone to subjectivity. As a result, consistency and reproducibility across practitioners are difficult to achieve. With continuous advancements in MRI technology and computational techniques, deep learning-based automated segmentation has emerged as a promising and rapidly expanding research frontier in KOA imaging [17,18,19,20]. Although previous AI-based studies have explored knee disease assessment using different imaging and non-imaging modalities, the present study focuses specifically on multi-target tissue segmentation in knee MRI.

**Figure 1:**
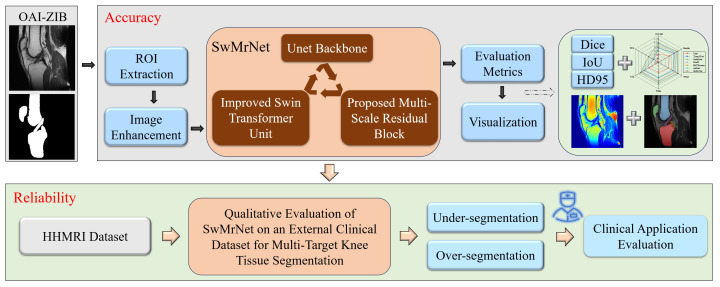
The flowchart of the proposed method. The OAI-ZIB dataset is used to quantitatively evaluate the segmentation accuracy of SwMrNet, the HHMRI dataset is employed for qualitative external clinical evaluation, and heatmaps are utilized to visualize the prediction confidence of SwMrNet.

Therefore, this paper introduces SwMrNet: a multi-target tissue segmentation method for robust and accurate clinical knee diagnosis assistance. This method significantly improves segmentation accuracy and robustness by integrating an enhanced Swin Transformer and a proposed multi-scale residual module. The improved Swin Transformer is primarily used to extract global features, capture the overall structure of tissues, and enhance the model’s generalization capabilities, while the proposed multi-scale residual module is designed to capture local detail features, refine tissue segmentation boundaries, and enhance segmentation precision. Experimental results on the publicly available Osteoarthritis Initiative-Zuse Institute Berlin (OAI-ZIB) dataset and the local clinical Honghui Hospital knee MRI (HHMRI) dataset demonstrate that SwMrNet achieves high-precision multi-tissue segmentation on the OAI-ZIB dataset, while the segmentation results on HHMRI were qualitatively reviewed by clinical experts. These findings underscore the model’s robust generalization ability, offering an efficient multi-target segmentation tool for the automated diagnosis of knee joint diseases in clinical practice. The flowchart of the proposed method is shown in Figure 1. The main contributions of this study are summarized as follows:

1. To meet the clinical need for automated knee joint tissue segmentation, a multi-target tissue segmentation method, SwMrNet, is proposed for knee MRI analysis and auxiliary clinical assessment.
2. SwMrNet integrates an improved Swin Transformer unit and a proposed multi-scale residual module into a U-Net-based framework, enabling complementary modeling of contextual information and local anatomical details to improve segmentation accuracy.
3. Experiments on the OAI-ZIB dataset demonstrate that SwMrNet achieves high segmentation accuracy in multi-target tissue segmentation. Qualitative evaluation on the local HHMRI dataset further shows visually consistent segmentation results, suggesting its potential applicability to clinical knee MRI analysis.

The remainder of this paper is organized as follows. Section 2 reviews the related work. Section 3 briefly introduces SwMrNet, the improved Swin Transformer unit, the proposed multi-scale residual block, and the evaluation metrics. Section 4 describes the experimental setup. Section 5 presents the experimental results. Section 6 discusses the main findings. Finally, Section 7 concludes the paper.

## 2. Related Work

Deep learning algorithms have demonstrated significant advantages in feature recognition and target segmentation of MRI images and have been widely applied in the precise segmentation of knee joint tissues. Based on the number of segmentation targets, knee joint segmentation can be categorized into single-target and multi-target segmentation.

In single-target segmentation, research primarily focuses on the segmentation of cartilage [21,22,23], while other structures, such as the meniscus and femur, have also become key areas of study. Wang et al. [24] proposed the use of patch attention U-Net for knee cartilage segmentation in MRI images, utilizing the patch attention block to capture intra-channel and intra-patch relationships. This method achieved high cartilage segmentation accuracy on the SKI-10 and OAI datasets. Reza et al. [25] employed an integrated 3D SwinUnet approach for automatic cartilage segmentation, significantly enhancing the finite element modeling accuracy for knee cartilage segmentation across both general and specific subject groups. Gait prediction experiments indicated that there was no significant difference in the finite element modeling accuracy and mechanical performance between automatic and manual segmentation. Anita et al. [26] introduced a specialized network model for meniscus segmentation, a depthwise convolutional neural network U-Net combined with a depthwise residual network. This method demonstrated superior clinical segmentation capabilities on 3T fat-suppressed turbo spin-echo MRI data. Daniel et al. [27] developed an enhanced attention U-Net method incorporating the Tversky loss function and deep layer structures to improve the model’s ability to extract key features. This approach achieved high-precision femur segmentation and 3D model construction. Experimental results showed that clinicians could visualize femoral changes in real time, thereby enhancing early intervention outcomes effectively.

Compared to single-target segmentation of the knee joint, multi-target segmentation offers a comprehensive structural view of the associated tissues, allowing doctors to evaluate the interactions between different tissues more holistically. This approach helps prevent the oversight of correlated pathologies that may be missed in single-target segmentation [27,28,29,30]. Wang et al. [20] proposed a deep learning approach that integrates PDWI segmentation results with patient data to classify common knee synovitis. Experimental results demonstrated that this method achieved an overall accuracy of 86%, significantly surpassing the classification performance of radiologists and effectively aiding in the clinical diagnosis of synovitis. Iwasa et al. [31] developed a deep learning model for the segmentation of the musculoskeletal system and hip from CT images. The model was tested on a large CT database, encompassing various disease conditions and patient positions (standing and supine) from multiple devices. The results indicated that this method notably enhanced segmentation accuracy across all metrics and improved the reliability of individual musculoskeletal segmentations, enabling fully automated MSK segmentation. Liu et al. [32] introduced the dual-path double attention transformer based on CT images for the segmentation of the femur, tibia, patella, and fibula. Experimental results showed that this method outperformed nnU-Net, TransUNet, and 3D U-Net models in segmentation performance and demonstrated superior accuracy compared to manual segmentation in robot-assisted total knee arthroplasty procedures. Although recent studies have explored advanced architectures for knee MRI segmentation [33,34,35], simultaneous segmentation of the femur, patella, tibia, anterior cruciate ligament, and posterior cruciate ligament remains less thoroughly investigated. Despite the notable advancements in knee joint segmentation presented in the aforementioned studies, several challenges persist in clinical applications. Clinical disease diagnosis requires collaborative detection of multiple tissues, and existing multi-target segmentation methods often suffer from low accuracy and poor generalization to clinical multi-modal knee MRI data, frequently necessitating retraining.

## 3. Methods

### 3.1. Multi-Target Tissue Segmentation Network of SwMrNet

**Figure 2:**
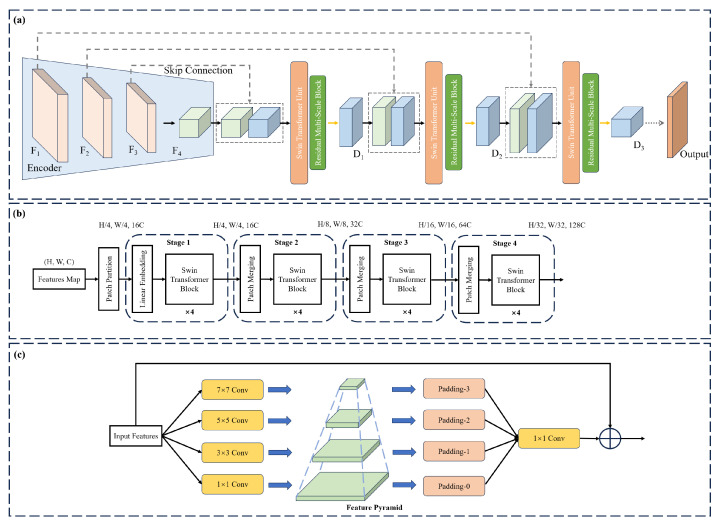
Multi-target tissue segmentation network of SwMrNet. (**a**) SwMrNet network flowchart; (**b**) improved Swin Transformer unit; (**c**) proposed multi-scale residual block.

In the clinical task of multi-target tissue segmentation of knee joints, enhancing both segmentation accuracy and robustness remains a substantial challenge. As a self-adaptive medical image segmentation framework, nnU-Net automatically optimizes the network architecture and training pipeline based on specific task requirements and data characteristics, exhibiting strong generalization capability and broad applicability. Building on its embedded U-Net architecture, this study incorporates an improved Swin Transformer unit and the proposed multi-scale residual module to strengthen the model’s global perception and local detail representation. Accordingly, the proposed segmentation network, SwMrNet, is developed. Furthermore, the input data are preprocessed following the standardized nnU-Net pipeline. The overall architecture of SwMrNet is depicted in Figure 2a.

The network architecture comprises an encoder and a decoder. The encoder is responsible for extracting multi-scale feature maps from the input image, thereby effectively capturing high-level semantic information. In contrast, the decoder progressively restores the spatial dimensions of the image and makes precise image segmentation predictions. The overall computation process of the network is as follows:

(1) Y=Decoder(Encoder(X)),

where $X\in R^{H\times W\times C_{in}}$ represents the input image; $Y\in R^{H\times W\times C_{out}}$ denotes the predicted output; *H* and *W* correspond to the height and width of the image; and $C_{in}$ and $C_{out}$ represent the number of input and output channels, respectively.

The encoder employs convolutional operations and downsampling to generate a set of feature maps $F_{i}$ at multiple scales. Each encoding stage $E_{i}$ consists of convolution, batch normalization, activation function, and max-pooling operations. Furthermore, the encoder uses skip connections to propagate feature maps from previous layers, thereby enhancing the image’s fine details while preserving spatial context. The computation for the *i*-th encoding stage is as follows:

(2) $$F_{i}=E_{i}\left(F_{i-1}\right),\;i=1,2,3,4;\;F_{0}=X.$$

The decoder is composed of multiple decoding stages, denoted as $D_{i}$, where each stage incorporates a Swin Transformer module along with decoding modules operating at multiple scales. Through the upsampling operation, the decoder progressively restores the spatial dimensions of the image, while integrating the encoder’s outputs to enable the stepwise recovery of the image. The computation for the *i*-th decoding stage and its subsequent processing via the Swin Transformer are expressed as follows:

(3) $$\left\{\begin{array}{c} D_{i}=UP\left(Concat\left(D_{i-1},F_{i-j}\right)\right),\;j=1,2,3;\;D_{0}=F_{4}\\ D_{i}^{\prime }=Re\left(sw\left(D_{i}\right)\right), \end{array}\right.$$

where UP represents the upsampling operation, Concat denotes the concatenation of feature maps; Re refers to the multi-scale residual module, and sw stands for the Swin Transformer.

### 3.2. Improved Swin Transformer Unit

The Swin Transformer module established in this paper is primarily designed to efficiently process global contextual features. The detailed process is illustrated in Figure 2b, and it consists of the following key steps:

(1) Patch partition (PP): The input feature map is partitioned into image patches of size 4×4, with each patch having a dimension of 16C. The calculation is as follows:
  (4) $$F_{patch}=PP\left(F_{in}\right),\;F_{patch}\in R^{\frac{H}{4}\times \frac{W}{4}\times 16C},$$
  where $F_{in}\in R^{H\times W\times C}$ represents the input feature map.
(2) Linear embedding (LE): Each image patch is then mapped to a linear representation, with the following formula:
  (5) $$F_{embed}=LE\left(F_{patch}\right).$$
(3) Swin Transformer block: This module consists of multiple consecutive transformer submodules, incorporating two types of W-MSA and SW-MSA exchanges. At each stage, the output feature map’s spatial dimensions are halved (patch merging), while the number of channels is doubled. The process is as follows:

(6) $$\left\{\begin{array}{c} Stage1:H\times W\times 16C,\\ Stage2:H\times W\times 32C,\\ Stage3:H\times W\times 64C,\\ Stage4:H\times W\times 128C. \end{array}\right.$$

The computation process for each Transformer submodule is

(7) $$\left\{\begin{array}{c} z^{l}=\mathrm{W-MSA}\left(LN\left(z^{l-1}\right)\right)+z^{l-1},\\ z^{l}=MLP\left(LN\left(z^{l}\right)\right)+z^{l},\\ z^{l+1}=\mathrm{SW-MSA}\left(LN\left(z^{l}\right)\right)+z^{l},\\ z^{l+1}=MLP\left(LN\left(z^{l+1}\right)\right)+z^{l+1}. \end{array}\right.$$

The final output feature map is given by

(8) $$F_{out}^{in}\in R^{H\times W\times 128C}.$$

### 3.3. The Proposed Multi-Scale Residual Block

The multi-scale residual block presented in this paper is specifically designed to capture local multi-scale features. The detailed process is illustrated in Figure 2c. It processes the input features by utilizing convolutional kernels of varying sizes, with the detailed computation process as follows:

(9) $$\left\{\begin{array}{c} F_{1\times 1}=Conv_{1\times 1}\left(F_{in}\right),\;padding=0,\\ F_{3\times 3}=Conv_{3\times 3}\left(F_{in}\right),\;padding=1,\\ F_{5\times 5}=Conv_{5\times 5}\left(F_{in}\right),\;padding=2,\\ F_{7\times 7}=Conv_{7\times 7}\left(F_{in}\right),\;padding=3. \end{array}\right.$$

Subsequently, the outputs from the convolutions of different sizes are concatenated to form multi-scale features, and the corresponding calculation formula is provided below:

(10) $$F_{\mathrm{multi-scale}}=Conv_{1\times 1}\left(Concat\left(F_{1\times 1},F_{3\times 3},F_{5\times 5},F_{7\times 7}\right)\right).$$

Finally, the output feature map is refined through residual connections, with the corresponding calculation formula as follows:

(11) $$F_{out}=F_{in}+F_{mulsca}.$$

### 3.4. Evaluation Metrics

To evaluate the segmentation accuracy of the SwMrNet model, this paper employs the Dice coefficient, Intersection over Union (IoU), and Hausdorff Distance at the 95th percentile (HD95) as evaluation metrics. These metrics are used to assess the multi-target tissue segmentation results for both modalities, where HD95 represents the 95th percentile of the Hausdorff Distance. The calculation methods for Dice, IoU, and HD are outlined as follows:

(12) $$Dice=\frac{2\times |U\cap T|}{\left|U|+|T\right|},$$

(13) $$IoU=\frac{\left|U\cap T\right|}{\left|U\cup T\right|},$$

(14) $$HD=\max\left(\underset{u\in U}{\sup}\underset{t\in T}{\inf}∥u-t∥,\underset{t\in T}{\sup}\underset{u\in U}{\inf}∥u-t∥\right).$$

where *U* is the predicted boundary, *T* is the gold standard boundary, ∥u−t∥ is the Euclidean distance between the points u∈U and t∈T, and sup and inf represent the supremum and infimum, respectively.

## 4. Experimental Setup

### 4.1. Datasets: OAI-ZIB and HHMRI

All clinical knee MRI scans used in this study were fully anonymized prior to analysis. All procedures were conducted in accordance with the ethical principles outlined in the Declaration of Helsinki. The study protocol was reviewed and approved by the Institutional Ethics Committee of Honghui Hospital, Xi’an Jiaotong University (Approval No. 202409018). Given the retrospective nature of the study, the requirement for informed consent was waived by the ethics committee.

This study utilizes two knee MRI datasets for experimental evaluation: the OAI-ZIB dataset and a local clinical dataset, HHMRI. These datasets differ in imaging equipment and acquisition parameters and are used to evaluate the segmentation accuracy and clinical applicability of the proposed SwMrNet model in multi-target tissue segmentation tasks. Specifically, the OAI-ZIB dataset is divided into training and validation sets in a 7:3 ratio, while the HHMRI dataset is dedicated to qualitative evaluation in real-world clinical scenarios. The OAI-ZIB dataset, widely used in osteoarthritis research, comprises 507 knee MRI scans acquired using the dual echo steady state (DESS) sequence. In this study, 50 patients were randomly selected, and four slices were extracted from each patient, resulting in a total of 200 images for manual annotation. The labeled anatomical structures include the femur, patella, tibia, anterior cruciate ligament, and posterior cruciate ligament. The reference masks were manually annotated by two clinical experts according to visible anatomical boundaries on the knee MRI scans. The final masks were generated by merging the annotations from the two experts, followed by review and correction of uncertain boundaries to obtain consensus reference masks. Formal inter-observer agreement was not assessed in this study. The HHMRI dataset was collected at Honghui Hospital. It consists of 30 knee MRI cases acquired using the SPAIR sequence between October and November 2024, including 15 male and 15 female subjects aged 50 to 72 years. For each HHMRI case, four slices were selected according to anatomical coverage and image quality, with priority given to slices containing as many of the five target structures as possible. This resulted in 120 fully anonymized images. HHMRI was used only for qualitative external clinical evaluation. Since pixel-level annotations were unavailable and selected slices were used, potential selection bias may exist. Future work will include full-volume annotation or systematic slice sampling for objective validation. Detailed dataset specifications are summarized in Table 1.

### 4.2. Implementation Details and Experimental Settings

All experiments were implemented within the nnU-Net v2 framework, with the proposed SwMrNet serving as the backbone segmentation network. The 2D full-resolution configuration of nnU-Net was adopted in this study. The OAI-ZIB dataset was randomly split at the patient level into training and validation sets with a ratio of 7:3, whereas the HHMRI dataset was used exclusively for external robustness evaluation. The input data were preprocessed according to the nnU-Net planning and preprocessing pipeline, including image format conversion, foreground cropping, intensity normalization, resampling, and patch-based sampling. Based on the automatically generated nnU-Net plan, the target spacing and patch size were set to [0.33,0.33] and [512,512], respectively. The batch size was set to 12, and the foreground oversampling ratio was set to 0.33. During training, SwMrNet was optimized using stochastic gradient descent with an initial learning rate of 0.01, a momentum of 0.99, Nesterov acceleration, and a weight decay of $3\times 10^{-5}$. A polynomial learning-rate scheduler was employed to gradually decay the learning rate throughout training. The model was trained for 250 epochs, with 500 training iterations and 50 validation iterations per epoch. The loss function consisted of Dice loss and cross-entropy loss, with equal weights assigned to the two terms. Deep supervision was enabled during training, and the loss weights assigned to auxiliary outputs were exponentially decreased with decreasing resolution. The data augmentation strategy followed the nnU-Net training pipeline, including random rotation, scaling, Gaussian noise, Gaussian blur, brightness adjustment, contrast adjustment, simulated low-resolution augmentation, gamma correction, and mirroring. During inference, deep supervision was disabled. Sliding-window prediction was adopted with Gaussian weighting and mirroring-based test-time augmentation. The predicted segmentation maps were resampled back to the original image space. No additional morphology-based post-processing was applied. For a fair comparison, all baseline methods, including U-Net, TransUNet, SwinUNet, MedT, BATFormer, and nnU-Net, were trained and evaluated using the same experimental settings as SwMrNet.

## 5. Results and Analysis

### 5.1. Ablation Study on the OAI-ZIB Dataset

To evaluate the actual contributions of the improved Swin Transformer unit and the proposed multi-scale residual module, a series of ablation studies was conducted. For the improved Swin Transformer unit, ablation studies were performed on three key architectural components: the number of layers per stage, the number of stages, and the window size. As presented in Table 2, increasing the number of layers enhances the model’s feature representation capability; incorporating a deeper stage is essential for capturing high-level semantic information; and using a 7×7 window size provides a balanced trade-off between local detail extraction and global context modeling. These results demonstrate that the improved Swin Transformer unit improves performance across all evaluation metrics.

To evaluate the contribution of different modules to the segmentation performance of the proposed SwMrNet method, ablation studies were conducted by integrating the multi-scale residual block and the improved Swin Transformer unit. As shown in Table 3, the baseline model, U-Net, exhibited relatively low accuracy in multi-target tissue segmentation tasks. The proposed multi-scale residual block significantly enhanced the model’s feature representation capability, while the incorporation of the improved Swin Transformer unit notably strengthened the model’s ability to capture global features. By combining both modules, SwMrNet achieved optimal performance across all evaluation metrics, fully validating the complementarity and synergistic effectiveness of this structural design.

### 5.2. Segmentation Accuracy Comparison Experiment on the OAI-ZIB Dataset

**Figure 3:**
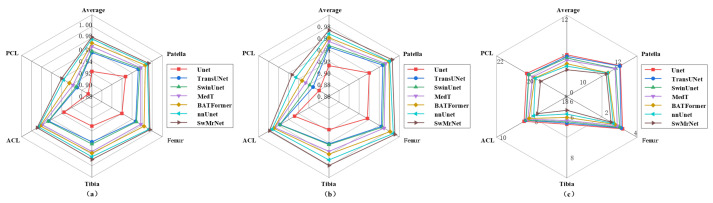
Radar chart of multi-target tissue segmentation comparison experiment on the OAI-ZIB dataset: (**a**) the Dice scores for segmentation; (**b**) the IoU scores for segmentation; (**c**) the HD95 scores for segmentation. Since a smaller HD95 value indicates higher segmentation accuracy, the scale ranges differ across different tissues.

To validate the segmentation performance of the proposed SwMrNet method, a series of comparative experiments was conducted, involving models such as U-Net, TransUNet, SwinUnet, MedT, BATFormer, and nnU-Net. As shown in Figure 3, SwMrNet demonstrates the best segmentation performance, achieving Dice and IoU scores of 98.2% and 97.4%, respectively, alongside the lowest HD95 value of 9.32. In contrast, U-Net exhibits the lowest segmentation accuracy. Figure 3a–c show that SwMrNet consistently outperforms nnU-Net in both overall performance and individual tissue segmentation, achieving the highest Dice and IoU scores and the lowest HD95 value. This reinforces the advantages of integrating the improved Swin Transformer unit with the multi-scale residual module, which significantly enhances segmentation accuracy. In the multi-target segmentation task, SwMrNet achieves Dice scores exceeding 98.15% for the patella, femur, tibia, and anterior cruciate ligament (ACL), with the posterior cruciate ligament (PCL) also achieving a Dice score of 94.04%. For a clearer comparison, the detailed per-class quantitative results are tabulated in Table 4. Segmentation performance varied across anatomical structures, with large bony structures being easier to segment due to clearer boundaries and larger regions, while cruciate ligaments, especially the PCL, remained more challenging because of their small size, low contrast, and partial volume effects. The quantitative results show that SwMrNet improves both region-overlap metrics, including Dice and IoU, and the boundary-sensitive HD95, indicating enhanced tissue-region consistency and boundary stability. This improvement may be attributed to the complementary decoder design of window-based contextual interaction and multi-scale residual refinement. These results indicate that SwMrNet performs effectively in multi-target tissue segmentation and may provide useful support for clinical diagnosis.

To further verify the statistical significance of the performance improvement, paired statistical analysis was performed based on the per-case segmentation results. The mean difference, standard error, 95% confidence interval, and *p*-value were calculated between SwMrNet and each baseline method. A positive mean difference indicates that SwMrNet outperformed the corresponding baseline method. As shown in Table 5, SwMrNet achieved positive mean differences compared with all baseline methods. The 95% confidence intervals of all comparisons were greater than zero, and the corresponding *p*-values were less than 0.05, indicating that the improvements of SwMrNet over the baseline methods were statistically significant.

**Figure 4:**
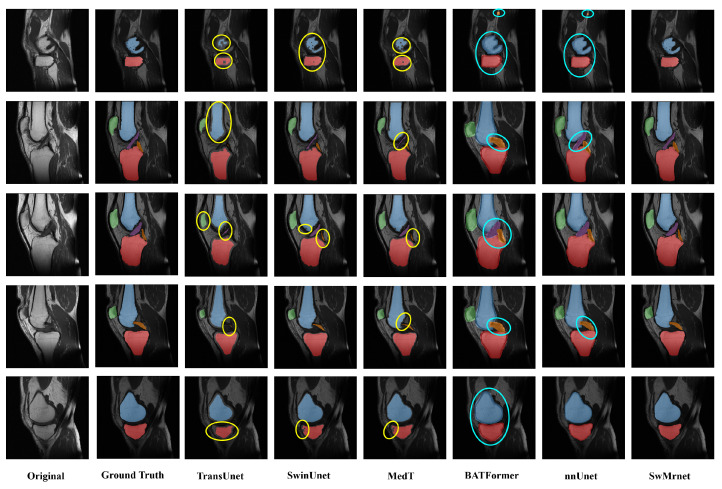
Visual comparison of segmentation results on the OAI-ZIB dataset. Different colors represent different anatomical structures, including the patella, femur, tibia, ACL, and PCL. The yellow circles indicate areas with noticeable under-segmentation issues, while the cyan circles highlight areas with noticeable over-segmentation issues, as identified by visual comparison with the corresponding ground-truth annotations.

To provide a clear comparison of segmentation performance across different methods, representative segmentation results were randomly selected and visualized. As shown in Figure 4, TransUNet, SwinUNet, and MedT exhibit noticeable under-segmentation in multi-target tissue segmentation, as indicated by the yellow circles, whereas BATFormer and nnU-Net show over-segmentation in several anatomically ambiguous regions, as highlighted by the cyan circles. These different error patterns may be related to insufficient local boundary recovery and the similar intensity distributions of adjacent tissues. In contrast, the proposed SwMrNet alleviates both under-segmentation and over-segmentation by introducing window-based feature interaction after decoder skip concatenation and applying multi-scale residual refinement to recover local anatomical details, thereby further validating the effectiveness of the proposed method.

**Figure 5:**
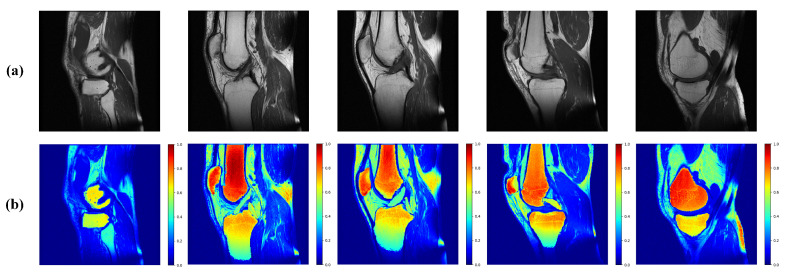
Heatmaps on the OAI-ZIB dataset: (**a**) the original image; (**b**) prediction confidence heatmaps generated from the final softmax probability maps of SwMrNet. Higher color values indicate higher prediction confidence for the corresponding tissue class.

To provide a qualitative visualization of the model prediction confidence in multi-target tissue segmentation, this study visualizes the pixel-wise probability maps generated by SwMrNet as heatmaps. These heatmaps were obtained from the final softmax output of the segmentation network, where higher values indicate higher prediction confidence for the corresponding tissue class. It should be noted that these heatmaps are not derived from Transformer attention weights and therefore should not be interpreted as direct evidence of the internal attention mechanism of the model. Instead, they provide a qualitative visualization of the spatial distribution of prediction confidence, which may help readers intuitively observe whether the model assigns high-confidence predictions to anatomically relevant regions. As illustrated in Figure 5, the model predominantly focuses on the patella, femur, tibia, cruciate ligaments, and regions exhibiting high-fat-signal intensity during the segmentation of knee joint tissues. These areas closely align with the signal categories observed in clinical practice. Consequently, clinicians can use SwMrNet for tissue segmentation and rely on the heatmaps to assist in diagnosis.

### 5.3. Qualitative External Clinical Evaluation on the HHMRI Dataset

**Figure 6:**
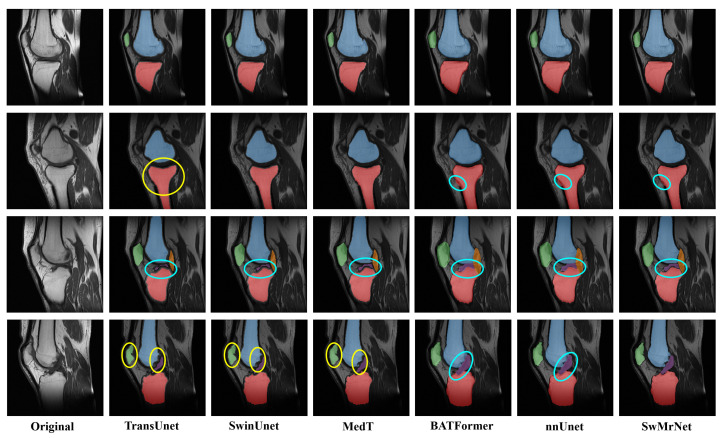
Visual comparison of segmentation results on the HHMRI dataset. Different colors represent different anatomical structures, including the patella, femur, tibia, ACL, and PCL. The yellow circles indicate areas with noticeable under-segmentation issues, as identified by clinical experts, while the cyan circles highlight areas with noticeable over-segmentation issues.

Since pixel-level annotations are unavailable for the HHMRI dataset, quantitative segmentation metrics could not be calculated on this external clinical dataset. The HHMRI dataset differs from OAI-ZIB in acquisition protocol, scanner vendor, magnetic field strength, and image size; specifically, OAI-ZIB consists of 3T DESS images, whereas HHMRI consists of 1.5T SPAIR images. Therefore, HHMRI was used as a challenging external clinical dataset for qualitative evaluation rather than objective performance comparison. To assess the clinical applicability and generalization potential of the proposed SwMrNet, the trained model was applied to the HHMRI dataset to generate multi-target tissue segmentation results. Representative cases were randomly selected and visualized to qualitatively compare the segmentation outputs of different methods. The visual assessment focused on anatomical plausibility, boundary continuity, and obvious segmentation failures, which are important factors for evaluating whether the segmentation results can assist clinical image interpretation. As shown in Figure 6, TransUNet, SwinUnet, and MedT exhibit significant under-segmentation issues, particularly in the serrated segmentation of the patella, as indicated by the yellow circles. In contrast, BATFormer and nnU-Net primarily suffer from over-segmentation, especially in redundant regions along the femur and tibia boundaries, with substantial erroneous segmentation areas in both the ACL and PCL, as highlighted by the cyan circles. The proposed SwMrNet demonstrates strong generalization capabilities, effectively addressing both under-segmentation and over-segmentation issues. Notably, in the case of PCL segmentation, where all models show misclassification, SwMrNet significantly reduces the erroneous segmentation regions. This further underscores that the proposed method outperforms others in terms of generalization and holds significant clinical application value, particularly for the automated segmentation and diagnosis of diseases related to the tibia, femur, and patella.

**Figure 7:**
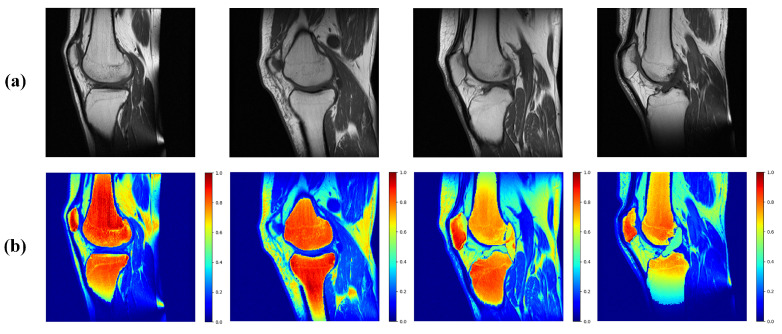
Heatmaps on the HHMRI dataset: (**a**) the original image; (**b**) prediction confidence heatmaps generated from the final softmax probability maps of SwMrNet. Higher color values indicate higher prediction confidence for the corresponding tissue class.

To improve the clinical interpretability of the SwMrNet segmentation results, this study visualizes the segmentation outcomes using heatmaps. As shown in Figure 7, the model primarily focuses on the patella, femur, tibia, low-signal ligaments, and high-intensity fat signal regions during the multi-target knee joint tissue segmentation. These areas are in close alignment with the signal categories observed in clinical practice. While the model exhibits some misidentification in low-signal regions, such as the PCL, its segmentation performance for other tissues is generally satisfactory. Consequently, clinicians can utilize SwMrNet for multi-target tissue segmentation, particularly for skeletal structures, and rely on the heatmap results to assist in diagnosis.

## 6. Discussion

Manual segmentation of knee joint tissues in clinical settings is often inefficient, and existing models frequently struggle with achieving accurate multi-target tissue segmentation. This study presents SwMrNet, an automatic segmentation network designed for knee MRI tissue segmentation and auxiliary KOA assessment. Built upon the U-Net architecture, SwMrNet integrates an improved Swin Transformer unit and a newly proposed multi-scale residual module to enhance segmentation accuracy across diverse anatomical structures. The model was evaluated on both the publicly available OAI-ZIB dataset and a local clinical dataset, HHMRI. Ablation experiments were conducted to investigate the contribution of individual architectural components and parameter configurations. Results show that the improved Swin Transformer unit and the proposed multi-scale residual module improved segmentation performance. On the OAI-ZIB dataset, SwMrNet was compared with several state-of-the-art segmentation models, including U-Net, TransUNet, SwinUnet, MedT, BATFormer, and nnU-Net. SwMrNet achieved superior results, with a Dice coefficient of 98.2%, IoU of 97.4%, and HD95 of 9.3. For individual structures, Dice scores exceeded 98.15% for the patella, femur, tibia, and ACL and reached 94.04% for PCL, highlighting the effectiveness of SwMrNet in multi-target tissue segmentation tasks. The prediction confidence heatmaps show that SwMrNet assigns high-confidence predictions to anatomically relevant regions, providing a qualitative visualization of model prediction confidence. Qualitative evaluation was further conducted on the HHMRI clinical dataset. The segmentation outputs were visually assessed with respect to under-segmentation and over-segmentation. SwMrNet showed visually more consistent segmentation results compared with competing methods, suggesting its potential applicability in clinical scenarios.

In conclusion, SwMrNet effectively reduces common issues such as under-segmentation and over-segmentation in knee joint tissue analysis. The network achieves high segmentation accuracy, offering a valuable solution for automated knee tissue segmentation and providing auxiliary support for computer-aided KOA assessment.

## 7. Conclusions, Limitations, and Future Work

This study introduces SwMrNet: a multi-target tissue segmentation method for robust and accurate clinical knee diagnosis assistance. The method demonstrates high segmentation accuracy and robustness in clinical practice, enabling the automatic segmentation of multiple knee joint tissues to a certain extent, thus holding significant clinical application value. However, several limitations remain. First, although SwMrNet achieved high accuracy in multi-target tissue segmentation on the OAI-ZIB dataset, the current study lacks an independent labeled external test set. The OAI-ZIB dataset was divided into training and validation sets, while the HHMRI dataset did not provide pixel-level annotations and was therefore used only for qualitative external clinical evaluation. As a result, objective quantitative metrics could not be calculated on HHMRI, and the external generalization ability of the model still requires further validation. Second, the current implementation performs segmentation from a single view, whereas knee MRI is commonly acquired from multiple views in clinical practice, such as coronal and axial planes. Multi-view information may provide complementary anatomical features and should be considered in future studies. Third, the proposed method focuses on multi-target tissue segmentation and does not directly integrate knee osteoarthritis diagnosis. Establishing a complete segmentation-assisted diagnostic framework would require extensive disease-level annotations and expert assessment from specialized clinicians. Fourth, a sex-stratified performance analysis was not conducted in this study. Since SwMrNet uses only MRI images as input and does not incorporate sex information, the current evaluation focused on overall segmentation performance. Future work will focus on constructing independent labeled external test sets, performing full-volume annotation of clinical MRI data, developing multi-view segmentation and diagnosis-assisted models, and conducting subgroup analyses to further improve the clinical applicability of automated knee tissue segmentation.

## Figures and Tables

**Table 1:** Details of the OAI-ZIB and HHMRI datasets.

Dataset	Images	Size	Strength	Sequence	Brand	Labels
OAI-ZIB	200	384 × 384	3T	DESS	Siemens	Yes
HHMRI	120	512 × 512	1.5T	SPAIR	Philips	No

**Table 2:** Ablation study results on the improved Swin Transformer unit.

Ablation Study	Layers	Stages	Size	Dice (%)	IoU (%)	HD95
Fewer Layers	(2,2,2,2)	4	7×7	97.88	96.82	9.49
More Layers	(6,6,6,6)	4	7×7	98.14	97.33	9.31
Our’s + No Deep Stage	(4,4,4,−)	2	7×7	98.06	97.19	9.32
Fewer Layers + No Deep Stage	(2,2,2,−)	3	7×7	97.86	96.79	9.49
More Layers + No Deep Stage	(6,6,6,−)	2	7×7	98.07	97.16	9.32
Small Window	(4,4,4,4)	4	3×3	97.84	96.77	9.49
Small Window	(4,4,4,4)	4	5×5	97.96	96.93	9.49
Large Window	(4,4,4,4)	4	9×9	98.12	97.28	9.31
Ours	(4,4,4,4)	4	7×7	98.15	97.37	9.31

Note: Layers denotes the number of layers in each stage. Stages refers to the total number of stages in the network. Size indicates the attention window size used in each stage. Dice, IoU, and HD95 represent the average values of these evaluation metrics across all multi-target tissue segmentation tasks.

**Table 3:** Ablation study results on the proposed SwMrNet.

Model	Dice (%)	IoU (%)	HD95
U-Net	92.24	91.27	10.05
U-Net + Mr	95.27	94.02	9.98
U-Net + Sw	96.68	95.97	9.60
Ours	98.15	97.37	9.31

Note: Mr represents the proposed multi-scale residual block, while Sw represents the improved Swin Transformer unit.

**Table 4:** Per-class quantitative comparison of different segmentation methods on the OAI-ZIB dataset.

Structure	Metric	U-Net	TransUNet	SwinUnet	MedT	BATFormer	nnU-Net	SwMrNet
**Patella**	**Dice (%)**	94.71	97.24	97.55	97.72	98.64	99.02	99.28
	**IoU (%)**	94.02	96.71	97.05	97.28	97.93	98.08	98.58
	**HD95**	12.04	12.01	11.78	11.74	11.34	11.32	11.24
**Femur**	**Dice (%)**	93.98	96.72	96.88	97.74	98.39	99.40	99.56
	**IoU (%)**	93.65	96.42	96.66	96.97	98.17	98.81	99.13
	**HD95**	3.17	3.09	3.01	2.87	2.74	2.64	2.51
**Tibia**	**Dice (%)**	93.17	95.89	96.27	97.55	97.83	98.44	98.93
	**IoU (%)**	91.74	94.19	94.33	95.49	95.97	96.96	97.91
	**HD95**	6.36	6.30	6.23	6.18	6.03	5.86	5.67
**ACL**	**Dice (%)**	93.55	96.62	96.74	97.88	98.29	98.63	98.91
	**IoU (%)**	92.86	95.76	95.82	96.78	96.99	97.33	97.87
	**HD95**	8.44	8.33	8.32	8.23	8.15	7.89	7.68
**PCL**	**Dice (%)**	88.74	90.94	91.15	91.77	92.48	93.62	94.04
	**IoU (%)**	87.96	89.17	89.84	90.65	91.37	92.55	93.37
	**HD95**	20.28	20.17	20.16	19.94	19.83	19.79	19.50
**Average**	**Dice (%)**	92.24	95.48	95.72	96.53	97.13	97.82	98.15
	**IoU (%)**	91.27	94.45	94.74	95.43	96.09	96.75	97.37
	**HD95**	10.06	9.98	9.89	9.79	9.62	9.45	9.31

**Table 5:** Statistical analysis of Dice scores between SwMrNet and baseline methods.

Baseline Method	Mean Difference ± SE	95% CI	*p*-Value
U-Net	5.41±0.19	[4.92,5.91]	p<0.05
TransUNet	2.66±0.17	[2.22,3.11]	p<0.05
SwinUnet	2.36±0.15	[1.98,2.74]	p<0.05
MedT	1.61±0.17	[1.17,2.05]	p<0.05
BATFormer	1.02±0.14	[0.65,1.39]	p<0.05
nnU-Net	0.32±0.05	[0.20,0.45]	p<0.05

## Data Availability

The datasets used and/or analyzed during the current study are available from the corresponding author, Liqiang Zhi, upon reasonable request.
